# Superinfection Exclusion in Neotropical Honey Bees May Block DWV‐B, an Emerging Infectious Disease Variant of Deformed Wing Virus

**DOI:** 10.1111/eva.70143

**Published:** 2025-08-12

**Authors:** Fernando A. Fleites‐Ayil, Claudia A. Castillo Carrillo, Luis A. Medina‐Medina, José Javier G. Quezada‐Euán, Hassan Shafiey, Robert J. Paxton

**Affiliations:** ^1^ Institute for Biology Martin Luther University Halle‐Wittenberg Halle (Saale) Germany; ^2^ Departamento de Apicultura Tropical, Facultad de Medicina Veterinaria y Zootecnia Universidad Autónoma de Yucatán Mérida Yucatán México; ^3^ German Centre for Integrative Biodiversity Research (iDiv) Halle‐Jena‐Leipzig Leipzig Germany

**Keywords:** Africanized honey bee, competitive exclusion, DWV‐A, DWV‐B, epidemiological modelling, prevalence, Yucatan peninsula

## Abstract

RNA viruses often comprise multiple variants that co‐circulate in a host population, with potentially complex dynamics. Deformed wing virus (DWV), arguably the most impactful virus of honey bees ( *Apis mellifera* ), nowadays exists as two major variants, genotypes A (DWV‐A) and B (DWV‐B), which provide an amenable window into the dynamics of multi‐variant pathogens. DWV‐B has increased in prevalence over the past two decades in honey bees in Europe, largely replacing DWV‐A. DWV‐B arrived over a decade ago in the New World, where its prevalence has also increased markedly in temperate North American honey bees. The Yucatan Peninsula of Mexico is home to a high density of both managed and feral Africanized honey bees (AHBs), which are also known to be infected by DWV, though variant dynamics in this tropical location have not been explored. Here, we present two temporally separated datasets on viral prevalence that demonstrate the presence of both DWV genotypes in Yucatecan AHBs in 2010, though with surprisingly little change in the high prevalence of DWV‐A and low prevalence of DWV‐B through to 2019. Epidemiological modeling suggests that the dynamics of DWV genotypes in AHBs of Yucatan may be due to a form of superinfection exclusion (SIE). We model one potential form of SIE, inter‐genotype recombination meltdown. In addition to providing information on the epidemiology of a major honey bee virus in the Neotropics, our results provide broader insight into the evolutionary dynamics of viruses that comprise two or more co‐occurring variants.

## 1 Introduction

RNA viruses exhibit high rates of mutation that can lead to their rapid evolutionary dynamics and emergence within a host population (Holmes 2009). They may comprise multiple variants that co‐circulate in a host population, with potentially complex dynamics that can impact the health of their hosts, be they wildlife, domestic species, or humans. During the recent COVID‐19 pandemic, variants of the SARS‐CoV‐2virus were replaced in a short lapse of time within human populations, for example, from alpha to delta and thence to omega (Li et al. 2021). Replacement events, whereby one viral variant replaces another, might be common to other viruses, though the mechanisms of replacement likely differ among viruses. Superinfection, wherein a host is infected by two or more pathogens or their variants (Schmid‐Hempel 2011), typically leads to displacement of the competitively inferior by the competitively superior variant and could thereby account for such replacement events, though the outcome of superinfection varies across host‐pathogen systems (Bashey 2015). Indeed, pathogen populations may retain multiple variants over time in a host population, as in dengue virus (DENV) infecting humans (Yadouleton et al. 2024), with potential consequences for disease virulence (Read and Taylor 2001; Rigaud et al. 2010; Alizon et al. 2013; Makau et al. 2022).

The western honey bee ( *Apis mellifera* L.) is considered one of the most important managed animals worldwide for its pollination services as well as honey and other hive products (Osterman et al. 2021). Consequently, *A. mellifera* has been traded widely, leading to its quasi‐global distribution (Beaurepaire et al. 2020). Its parasites and pathogens have similarly achieved near‐worldwide distribution, as in the case of its ectoparasitic mite *Varroa destructor* Anderson & Trueman, 2020, exotic to *A. mellifera* (Traynor et al. 2020), and numerous viruses (Beaurepaire et al. 2020), for some of which *V. destructor* (varroa) acts as an important vector (Yañez et al. 2020). Among the viruses closely associated with honey bees and vectored by varroa, *Iflavirus aladeformis* (deformed wing virus or DWV) in particular has risen to prominence (Grozinger and Flenniken 2019; Martin and Brettell 2019) through its close association with varroa ectoparasitism (Martin et al. 2012; Doublet et al. 2024), its worldwide spread (Wilfert et al. 2016), and because it has been closely linked to colony decline and loss, particularly in temperate regions of the Northern Hemisphere (Highfield et al. 2009; Dainat et al. 2012; Francis et al. 2013; Natsopoulou et al. 2017; Claing et al. 2024).

DWV, a Picorna‐like +(ss)RNA virus in the family *Iflaviridae*, is nowadays found as two common variants, the longstanding and widespread genotype A (DWV‐A; Wilfert et al. 2016) and the more recently described genotype B (DWV‐B; Ongus et al. 2004). Since the first description of DWV‐B (synonym *Varroa destructor* virus–1) isolated from *V. destructor* and *A. mellifera* in the Netherlands in 2001 (Ongus et al. 2004), it has subsequently spread around the globe (Paxton et al. 2022). This is presumably due to its high rate of transmission, which may be attributed to its higher rate of replication in host bees (e.g., McMahon et al. 2016; Ray et al. 2021; Norton et al. 2024) as well as its potentially more efficient vectoring by *V. destructor* in comparison to DWV‐A; whilst both genotypes of DWV are transmitted between honey bees primarily by the vector *V. destructor* , DWV‐B can additionally replicate within *V. destructor* (‘biological vectoring’: Gusachenko et al. 2020; Gisder and Genersch 2021) whereas DWV‐A apparently cannot (‘mechanical vectoring’: Posada‐Florez et al. 2019; but see Damayo et al. 2023).

In the Northern Hemisphere, DWV seems to exhibit an evolutionary dynamic in which one variant replaces another, akin to the dynamics of SARS‐CoV‐2in humans (Li et al. 2021). For example, DWV genotype C seems to have recently disappeared from UK honey bees (Kevill et al. 2019), potentially having been replaced by DWV‐A and, subsequently, by DWV‐B (Paxton et al. 2022). Moreover, DWV‐D, exhumed from Egyptian honey bees collected in the 1960s, is seemingly absent from extant host honey bee populations (de Miranda et al. 2022), potentially having been replaced by DWV‐A or DWV‐C. Currently, DWV‐B seems to be replacing DWV‐A in many honey bee populations across the world (Paxton et al. 2022). Despite the higher virulence of DWV‐B over DWV‐A in adult honey bees (McMahon et al. 2016) that could theoretically limit its spread, a putatively higher rate of transmission of DWV‐B over DWV‐A may explain why DWV‐B has increased markedly in prevalence in many northern temperate regions during the last decade (USA: Ryabov et al. 2017; UK: Kevill et al. 2021; Germany and Italy: Paxton et al. 2022). These data suggest that DWV‐B is competitively superior to DWV‐A in temperate regions of the world. The change in dominant genotype has applied relevance because DWV‐B has been shown to be more damaging than DWV‐A: it more rapidly kills adult honey bees (McMahon et al. 2016). The replacement of DWV‐A by DWV‐B might also have contributed to high rates of honey bee colony mortality in Europe over the past two decades (Osterman et al. 2021), and DWV‐B may have contributed to the high mortality of honey bee colonies recently reported in early 2025 in California (Lamas et al. 2025).

That DWV‐B has not only increased in prevalence within honey bee populations but has also largely replaced DWV‐A (e.g., Germany: Paxton et al. (2022); Switzerland: Maurer et al. (2024)) is more of an enigma. Epidemiological modeling suggests that inter‐genotype interference plays a role because, when there is no interaction between genotypes, the prevalence of both genotypes is predicted to rise (Paxton et al. 2022). However, data on viral prevalence collected over two or more time points suggest that, when both genotypes are present in a host population, DWV‐B entirely replaces DWV‐A through an undefined form of interference when hosts are co‐infected. Superinfection exclusion (SIE) has been coined for one form of inter‐genotype interference competition, whereby one virus variant may block the establishment of another when both variants co‐infect the same host (Labrie et al. 2010). Indeed, it has already been suggested that DWV exhibits SIE; DWV‐B has been hypothesized to block the establishment of DWV‐A in a British population of honey bees (Mordecai et al. 2016), though the mechanism of interference was not identified.

Recombination meltdown has recently been proposed (Paxton et al. 2022) as one potential mechanism of viral SIE, whereby high rates of recombination may lead to the elimination of one genotype and thereby improve the establishment of another. RNA viruses can be highly recombinogenic, potentially as a mechanistic byproduct of their RNA polymerase (RdRp) processivity (Holmes 2009; Simon‐Loriere and Holmes 2011). Recombinants between DWV‐A and DWV‐B have often been reported within populations of honey bees (UK: Moore et al. 2011; Wang et al. 2013; Ryabov et al. 2014; Israel: Zioni et al. 2011; Daughenbaugh et al. 2021; France: Dalmon et al. 2017; Tunisia: Abdi et al. 2018; Hawaii: Brettell et al. 2019, 2020; Spain: Barroso‐Arévalo et al. 2019; Turkey: Şevik et al. 2024; USA: Hesketh‐Best et al. 2024; Europe: Sircoulomb et al. 2025), suggesting that recombination occurs regularly in hosts co‐infected by both genotypes. Paxton et al. (2022) hypothesized that inter‐genotype SIE via recombination meltdown could explain the current replacement of DWV‐A by DWV‐B in Europe, though without mathematical support for the verbal argument. Indeed, Mordecai et al. (2016) originally hypothesized that recombination meltdown between genotypes A and B would lead to the blocking of DWV‐A by DWV‐B in their study of SIE in a British population of honey bees. Other forms of interference competition, e.g., priority effects (Bashey 2015), might play an alternative or additional role in DWV‐B's replacement of DWV‐A in temperate regions.

The Americas make for an interesting case to test the hypothesis that SIE dictates the dynamics of viral genotypes. Both DWV‐A and DWV‐B have been detected in many countries of the Americas (Paxton et al. 2022), though the recent detection at low prevalence of DWV‐B in host populations of honey bees infected at a high prevalence with DWV‐A suggests that DWV‐B is a recent arrival (Ryabov et al. 2017; Paxton et al. 2022). In the USA, honey bee populations have witnessed a dramatic increase in the geographic range and prevalence of DWV‐B since its first detection in 2010, including on the islands of Hawaii (2010–2019; Grindrod et al. 2021; Zhang et al. 2024) and mainland USA (2010–2016; Ryabov et al. 2017), where there was (in 2016) approximate parity in the prevalence of DWV‐A and DWV‐B. More recent sampling (2021) and whole genome (long‐read) sequencing to reveal honey bee and varroa mite viromes support ongoing replacement of DWV‐A by DWV‐B and further suggest that A‐B recombinants are widespread in Continental USA (Hesketh‐Best et al. 2024). The dynamics of DWV‐genotype replacement in temperate North American honey bees may therefore mirror that in Europe.

In tropical and subtropical regions of the Americas, Africanized honey bees (AHBs) provide even deeper insight into the dynamics of co‐occurring DWV genotypes and the SIE hypothesis because their populations are large and generally unmanaged to control varroa mites and the viruses they vector, including DWV (Düttmann et al. 2021; Guzmán‐Novoa et al. 2024). Furthermore, the prevalence of DWV in AHBs can be high (e.g., 97% in Costa Rica; Chaves Guevara et al. 2024). Data from tropical American countries is, though, currently limited to single time‐point estimates: Brazil (de Souza et al. 2019), Argentina (Brasesco et al. 2020; Gonzalez et al. 2024), Colombia (Tibatá et al. 2021), Chile (Riveros et al. 2020), and the Yucatan Peninsula of Mexico (Fleites‐Ayil et al. 2023). These studies nevertheless confirm the presence of DWV‐A and DWV‐B in AHBs, with DWV‐A being far more prevalent than B, presumably because DWV‐B has recently entered honey bee populations of these countries as part of its global expansion (Paxton et al. 2022). But they do not allow examination of the dynamics of viral genotypes, which may differ from those in temperate regions of the world, where DWV‐B seemingly excludes DWV‐A.

Here, we examine the dynamics of DWV genotypes in one tropical American region, the Yucatan Peninsula of SE Mexico, using an original dataset on DWV‐genotype prevalence in drone honey bees collected in 2010 as well as a published dataset of worker honey bees collected from the same region in 2019 (Fleites‐Ayil et al. 2023). We hypothesized that DWV‐B, if present for long enough, would have replaced DWV‐A, as already seen in temperate European populations of honey bees. Through epidemiological modeling, we then explore possible mechanisms to explain the dynamics of genotypes A and B under three scenarios: independent spreading of genotypes (no SIE), weak SIE through mutual inhibition of genotypes, and strong SIE through recombination meltdown. Our data and modeling provide insight into why DWV‐B generally, but not always, replaces DWV‐A in host populations, and may inform on variant replacement in other host‐virus systems exhibiting superinfection.

## 2 Materials and Methods

### 2.1 Sample Collection

The Yucatan Peninsula in tropical SE Mexico is considered one of Mexico's and the world's most important apicultural regions because of its high honey production from managed AHBs (Güemes‐Ricalde et al. 2003), which have dominated the region since their arrival in the late 1980s (Quezada‐Euán et al. 1996; Clarke et al. 2001). Nowadays, the Yucatan Peninsula has a very high density of both managed and feral AHB colonies (Moritz et al. 2013; Domínguez‐Ayala et al. 2016). Following the replacement of European‐descent honey bees by AHBs, *V. destructor* was first detected in the Yucatan Peninsula in 1994, where it is now widespread (Medina and Martin 1999) and presumably facilitating viral transmission. DWV was first reported from Mexican honey bees in 2012 (Guzmán‐Novoa et al. 2012). Elsewhere in the Neotropics, the colony‐level prevalence of DWV in AHBs is high (e.g., 97% in Costa Rica; Chaves Guevara et al. 2024), likely because neither managed nor feral colonies are regularly treated to reduce the number of vectoring *V. destructor* , as AHBs show tolerance to this ectoparasitic mite (Düttmann et al. 2021; Guzmán‐Novoa et al. 2024). It is likely that varroa mites and DWV are ubiquitous in Latin American honey bee colonies. Recently, we have shown that AHB workers sampled from the field in the Yucatan Peninsula in 2019 were infected with both DWV‐A and DWV‐B, wherein DWV‐A was more prevalent (13%) than DWV‐B (2%) (Fleites‐Ayil et al. 2023).

**FIGURE 1:**
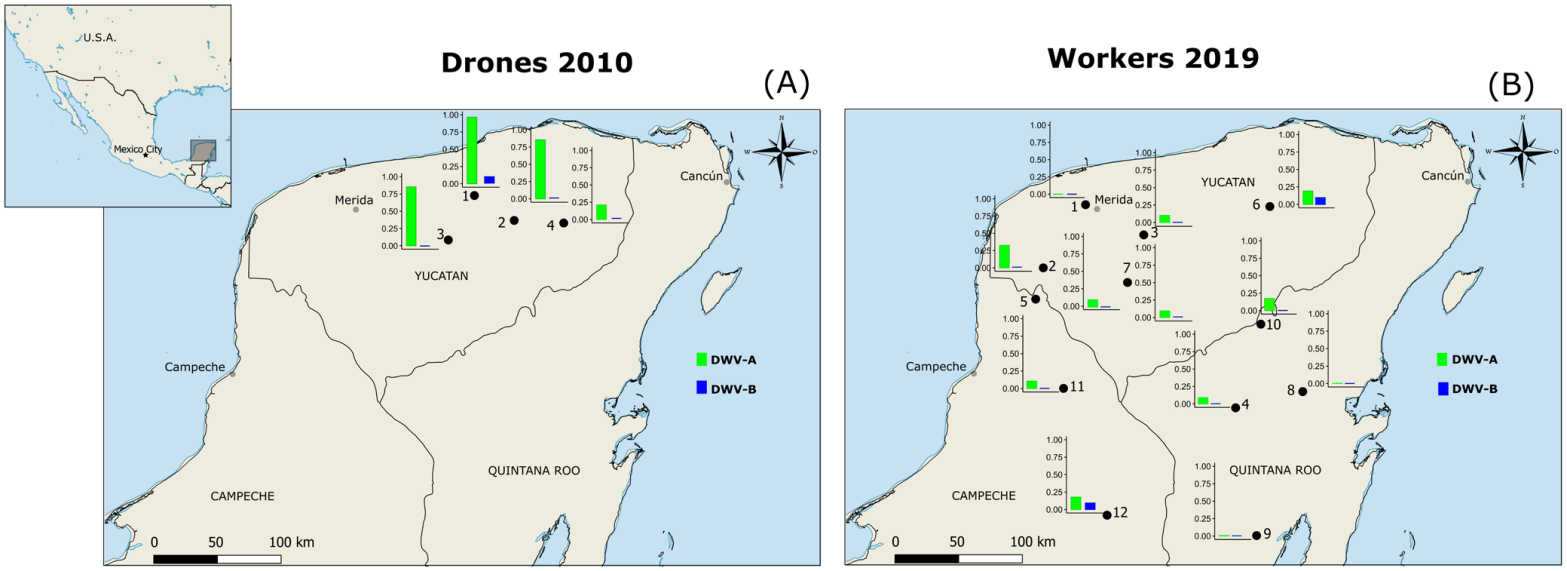
DWV‐genotype prevalence among honey bees of the Yucatan Peninsula, Mexico, in 2010 and 2019. (A) DWV‐A and DWV‐B prevalence in AHB drones collected from DCAs in 2010; (B) DWV‐A and DWV‐B prevalence in AHB workers collected from flowers in 2019. DWV‐A and DWV‐B are represented by green and blue bars, respectively; location names are in Table S1.

To allow us to explore the dynamics of DWV genotypes in AHBs of the Yucatan Peninsula, we complemented our 2019 DWV prevalence estimate in worker AHBs (collected from flowers at 12 sites across the Yucatan Peninsula: Fleites‐Ayil et al. (2023); Figure 1B, Table S1) with a retrospective analysis of drones collected in 2010 from four locations (Figure 1A, Table S1). Drones were captured in 2010 at drone congregation areas (DCAs) using an aerial trap baited with synthetic queen mandibular gland sex pheromone (E‐9‐oxo‐2‐decenoic acid; 12.5 μg/mL in 70% ethanol). The trap was raised to ca. 15 m above the ground using weather balloons filled with helium between 14:00 and 18:00 h and checked every 30 min to collect trapped drones (Jaffé et al. 2010). Each drone was separated into head, thorax, and abdomen, each stored individually in RNA‐Later (QIAGEN, Hilden, Germany), then transported to the laboratory, where samples were stored at −80°C until RNA extraction.

A full description of sampling in 2019 is given in Fleites‐Ayil et al. (2023). In short, from January to April 2019 during the flowering season, worker honey bees were sampled from 1000 m^2^ flower patches (dimensions 10 × 100 m or 20 × 50 m) at 12 locations (Figure 1B). To do so, we walked a continuous transect within a flower patch, collecting maximally 10 honey bees per 10 min until we had collected ca. 30 honey bees. Flower patches were embedded in a rural matrix comprising gardens, parks, and wildflower meadows. Bees were transferred immediately to individual 1.5 mL vials filled with RNA‐Later (QIAGEN, Hilden, Germany) and maintained in the field on dry ice (ca. −80°C) to avoid RNA degradation (Human et al. 2013) before storage at −80°C until RNA extraction.

### 2.2 RNA Extraction and Viral Detection

To detect variants of DWV, we extracted RNA from bees individually, using abdomens of drones or whole bodies of workers, using the methods of Tehel et al. (2019). Individual samples were crushed in 500 μL RLT‐buffer containing 1% β–mercaptoethanol using a plastic pestle, from which total RNA was extracted using an RNeasy Mini kit (QIAGEN, Hilden, Germany) in a QIAcube extraction robot (QIAGEN) and eluted into 30 μL RNAse‐free water. cDNA was synthesized in a 10 μL volume from 800 ng of RNA using Oligo‐dT oligonucleotides (Thermo Scientific) and reverse transcriptase (M‐MLV and Revertase, Promega, Mannheim, Germany) following the manufacturers' instructions, then diluted 1:10 before use in qPCRs.

In total, 250 drones were initially screened in 2010 by RT‐PCR (qPCR) (Table S1) using generic primers for DWV that do not differentiate between DWV genotypes (Table S2). A subset of 89 drones from the four sampling locations that were positive for DWV was then screened for DWV‐A and DWV‐B using genotype‐specific primers (Table S2), as were all 114 workers collected in 2019 (Table S1). We performed duplicate qPCR reactions per sample in a Bio‐Rad C1000 thermal cycler (Bio‐Rad, Munich, Germany) using SYBR green Sensimix with the following program: 5 min at 95°C, followed by 40 cycles of 10 s at 95°C, 30 s at the primer *t*_m_, and 30 s at 72°C.

We set the PCR cycle quantification (Cq) threshold at < 35 to consider a sample as positive for a viral target and averaged Cq values of technical duplicates. Two positive (extract of an infected bee) and two negative (template‐free) control wells were included per 96 well plate; they were consistently positive (Cq < 35) or negative (Cq > 35), respectively. We also ran a melt curve profile for each qPCR product in which PCR products were denatured for 1 min at 95°C, cooled to 55°C for 1 min, and then a dissociation (melt curve) profile was generated from 55°C to 95°C at an increment of 0.5°C per second to ensure a single product of the correct dissociation (‘melt’) temperature had been generated. We rejected samples that did not meet these criteria. We also qPCRed honey bee reference genes β‐actin (drones and workers) and ribosomal protein 49 (RP49: drones only) to ensure that RNA extraction, cDNA synthesis, and qPCRs were successful (primers given in Table S2); if technical duplicates of both reference genes differed by more than one cycle, we rejected the sample. The few samples for which both reference genes had a Cq > 30 were rejected. Data from the honey bees collected in 2019 are from Fleites‐Ayil et al. (2023).

### 2.3 Statistical Analysis

Statistical analyses and plots of field data of viral prevalence were undertaken in R v. 4.1.1 (R Core Team 2021). We calculated viral prevalence and 95% CIs per time point using the R package “epiR” v. 2.0.63 (Stevenson et al. 2017). In calculating the absolute prevalence of DWV genotypes in drones, we accounted for our subsampling of 89 drones from the initial 250 drones screened for DWV using DWV‐generic qPCR primers. Fisher exact tests (in the R package “stats”) were used to evaluate the statistical significance of changes in the absolute prevalence of DWV‐A and of DWV‐B as well as the change in relative prevalence (as the number of DWV‐positive bees with A or B) between the two time points of sampling (2010, 2019).

### 2.4 Phylogenetic Analysis of DWV‐A Sequences

Six DWV‐A positive samples, three drones from 2010 and three workers from 2019, were selected for sequencing to determine the phylogenetic relationship between Yucatecan isolates of DWV‐A and those from other regions of the world. To do so, we PCR amplified a 451 bp partial sequence of the RdRp gene using primers F15 (5′‐TCC ATC AGG TTC TCC AAT AAC GGA‐3′) and B23 (5′‐CCA CCC AAA TGC TAA CTC TAA GCG‐3′) (Yue and Genersch 2005) with the PCR conditions: 94°C for 2 min, followed by 35 cycles of 94°C for 30 s, 54.3°C for 1 min, and at 72°C for 30 s, and a final extension step at 72°C for 5 min. PCR products were purified using the QIAquick PCR Purification Kit (Qiagen, Hilden, Germany) and cloned directly using the pGEM T Easy Vector System II (Promega, Mannheim, Germany) following the manufacturer's instructions. Three randomly selected clones per sample were commercially Sanger sequenced in the forward and reverse directions (GATC Biotech, Constance, Germany), assembled into contigs, and aligned manually using Geneious v7.0.6 (Kearse et al. 2012) to a reference genome of DWV‐A (NC_004830).

Pruning identical sequences retrieved from an individual bee resulted in 11 unique sequences (5 from 2010 samples, 6 from 2019 samples), each with a length of 403 nucleotides, corresponding to the nucleotide positions 9280–9682 on the NCBI reference genome (NC_004830). The phylogenetic relationship among sequences was estimated using maximum likelihood in IQ‐TREE (Nguyen et al. 2015) with the HKY + F + G4 model, which had the optimal BIC score, as determined by ModelFinder (Kalyaanamoorthy et al. 2017). Bootstrap support for the tree was estimated using Ultrafast bootstrap approximation (Hoang et al. 2018) using 1000 replicates. The tree was visualized and annotated online using iTOL (https://itol.embl.de/). To determine if the DWV‐A variants were shared among these time points at our study area, we also generated a Median‐Joining haplotype network in PopART v1.7 (Leigh and Bryant 2015). In addition to our samples from Mexico, we also included the RdRp fragment from 12 complete genomes of DWV‐A from across the world as well as a DWV‐B reference genome (NC_006494) as an outgroup to investigate relationships among sequences.

### 2.5 Epidemiological Model Describing the Dynamics of DWV Genotypes

We here extend the deterministic epidemiological model first presented in Paxton et al. (2022) to describe the dynamics of DWV‐A and DWV‐B, with a focus on the case in which DWV‐B enters a host population with a pre‐existing high prevalence of DWV‐A, as found in the Yucatan Peninsula (see Section 3). In contrast to the previous ‘compartment’ (or SI—susceptible‐infectious) model in which each compartment (infected with A, infected with B, infected with A and B, uninfected) had a different fatality rate (Paxton et al. 2022), the current model focuses on the frequencies and fatality rates of different viral genotypes in a population of honey bees. In doing so, some of the model's equations are analytically solvable. The model runs in continuous time within an infinitely large honey bee population, likely approximating the very large AHB population in the tropical Yucatan Peninsula of Mexico (Moritz et al. 2013; Domínguez‐Ayala et al. 2016).

We first define a honey bee individual as being in one of the four states:

- H (healthy, accurately defined as uninfected by either genotype of DWV),
- A (infected only with DWV‐A),
- B (infected only with DWV‐B), or
- M (mixed), when co‐infected (by both DWV‐A and DWV‐B).

Symbols A and B (and corresponding lower case letters a and b) are used to indicate the viral genotypes A and B (and their corresponding frequencies *a* and *b*) in a population of honey bees. In addition, symbols *a*′ and *b*′ are used to indicate the frequency of DWV‐A and DWV‐B at equilibrium. We initiated simulations by setting the frequency of DWV‐B at 0.01 (*b* = 0.01) and different values (> 0.01) for the frequency of DWV‐A to reflect a population of honey bees in which DWV‐A initially predominates and is first invaded by DWV‐B, the most plausible real‐life scenario in the Yucatan Peninsula (see Section 3).

A viral genotype X (with the frequency of *x* in the population of honey bees) is described by two parameters: transmission rate (*μ*_*X*_), which is defined as the fraction of X‐uninfected individuals which will be infected in the next time step, conditional on the availability of infected individuals in the current time step; and their fatality rate (ν_X_), defined as the fraction of X‐infected individuals which will die in the next time step. We set *μ*_*B*_ > *μ*_*A*_, as suggested by the increasing prevalence of DWV‐B during the last decade in Europe and USA (Ryabov et al. 2017; Paxton et al. 2022), and the potential for DWV‐B to replicate in vectoring varroa mites (Gusachenko et al. 2020; Gisder and Genersch 2021) as well as to replicate faster in adult honey bees (McMahon et al. 2016) in comparison to DWV‐A.

The model incorporates the concept of SIE, in which infection by one pathogen can potentially limit the spread of a subsequent pathogen. There are two ways in which the frequency of genotype A in the population (denoted by *a*) can increase. Firstly, a can increase:

- When a healthy individual (uninfected by either DWV genotype) becomes infected with genotype A; this event occurs at the rate of *a* (1−*a*) (1−*b*) *μ*_*A*_.

In this expression, *a* is the fraction of the population infected by DWV‐A and which plays the role of the source of infection in this transmission event (*b* is the fraction of a population infected by DWV‐B). (1−*a*) (1−*b*) is the fraction of healthy individuals which play the role of sink (recipient) in the transmission event. Finally, *μ*_*A*_ is a constant which captures the rate of transmission of DWV‐A when a healthy individual is in contact with an infected individual. In honey bees, transmission of a virus may happen inside a colony when a nurse feeds larvae, when infected drones mate with the queen at a DCA, when an infected queen lays eggs, when two workers exchange food, or among workers when a virus is transmitted by phoretic varroa mites (Martin and Brettell 2019; Yañez et al. 2020). Though different transmission routes have their own transmission rates, for simplicity we do not distinguish among them. We thereby assign a constant value (*μ*_*A*_ and *μ*_*B*_) to capture the overall rate of transmission of DWV‐A and DWV‐B, respectively.

Secondly, the frequency of genotype A can also increase in the population:

- when a B‐infected individual becomes infected with genotype A; this event occurs at the rate of *a* (1−*a*) *bm*_A_.*μ*_A_.

In this expression, *a* is again the fraction of A‐infected individuals, (1−*a*) *b* is the fraction of individuals that are infected only by DWV‐B and *m*_*A*_.*μ*_*A*_ is the transmission rate. In this case, the transmission rate of genotype A is inhibited by genotype B, which is already present in the host; hence, it is reduced by a factor of *m*_*A*_, which captures the extent of this inhibition; the latter varies from 0 (complete inhibition) to 1 (no inhibition). In this model, we do not consider the case of cooperation, in which infection with one virus variant facilitates transmission of the other, as it is not consistent with the documented dynamics of DWV genotypes in Europe and the USA.

Additionally, the frequency of genotype A decreases when an A‐infected individual or a co‐infected individual dies; this event occurs at the rate of *a.ν*_*A*_, which depends only on the fatality rate of genotype A.

Symmetrically the same events happen to genotype B. The change in the frequency of genotypes A and B is then given by Model 1:

$$\text{Model}\;1:\left\{\begin{array}{c} \mathrm{da}/\mathrm{dt}=a\;\left(1-a\right)\;\left(1-b\right)\;\mu_{A}+\left(1-a\right).a.b.m_{A}\mu_{A}-a.\nu_{A}\\ \mathrm{db}/\mathrm{dt}=b\;\left(1-b\right)\;\left(1-a\right)\;\mu_{B}+\left(1-b\right).b.a.m_{B}.\mu_{B}-b.\nu_{B} \end{array}\right.$$

Though experimental evidence points to the higher virulence of DWV‐B over DWV‐A in adult honey bees (*ν*_*B*_ ≥ *ν*_*A*_; McMahon et al. 2016), conservatively we initially assume the fatality rate of both viral genotypes is the same (i.e., *ν*_*A*_ = *ν*_*B*_) as we seek to understand whether DWV‐B may be blocked by DWV‐A (see Section 3).

We explore the dynamics of this model under three different conditions (see Section 3): independent spreading of genotypes, mutual genotype inhibition (SIE), and inter‐genotype recombination meltdown, a potentially strong form of SIE.

As several of these equations are non‐linear, we used simulations with the package “deSolve” (Soetaert et al. 2010) in R v. 4.1.1 (R Core Team 2021) to describe the dynamics of the frequency of DWV genotypes in a population of honey bee individuals across plausible parameter space.

Figures from epidemiological modeling were generated in R v. 4.1.1 (R Core Team 2021).

## 3 Results

### 3.1 Viral Prevalence

Of the 250 drones originally sampled and successfully screened for DWV in 2010 using generic primers that do not differentiate between DWV genotypes, a mean of 66% (95% CI: 60%–72%) that passed our quality criteria were positive (*C*q < 35) for DWV (Figure S1, data in Table S3). Using genotype‐specific qPCR primers on 89 of these DWV‐positive samples, we found both DWV‐A and DWV‐B (Figure 1A, data in Table S4), albeit with DWV‐B at low prevalence and titer. DWV‐A was found in all 89 DWV‐positive drones, whilst one drone was also infected with DWV‐B (mean percent prevalence 0.7%, 95% CI: 0.02%–4%). Its Cq value suggested a high DWV‐A titer and a low DWV‐B titer (Figure S2; data in Table S4).

In 114 workers sampled from flowers in 2019, DWV‐A had a mean percent prevalence of 12% (95% CI: 7%–19%), while that of DWV‐B was 2% (95% CI: 0.2%–6%; Figure 1B). The two workers positive for DWV‐B did not exhibit a qPCR signal for DWV‐A.

Between 2010 and 2019 in Yucatecan honey bees, there was a significant drop in the absolute prevalence of DWV‐A (Fisher's exact test *p* = 0.0001), while the low absolute prevalence of DWV‐B did not change (Fisher's exact test *p* = 0.5944; Figure S3). Thus, in contrast to data from Europe and North America, DWV‐B has not risen rapidly in prevalence in AHBs of the Yucatan Peninsula, and DWV‐A has not seemingly been replaced over time; DWV‐B has remained at low prevalence (Figure 1, Figure S3).

There was a subtle decrease in the relative prevalence of A versus B among DWV‐infected bees between 2010 and 2019 (Fisher's exact test *p* = 0.0589; Figure S3), suggesting an overall decrease in DWV‐A's prevalence. However, this, as well as the difference in absolute prevalence of DWV‐A between 2010 and 2019, may reflect the inherent susceptibility or exposure of drones (collected at DCAs in 2010) versus workers (collected at flowers in 2019) to DWV.

### 3.2 Phylogenetic Relations of DWV‐A

**FIGURE 2:**
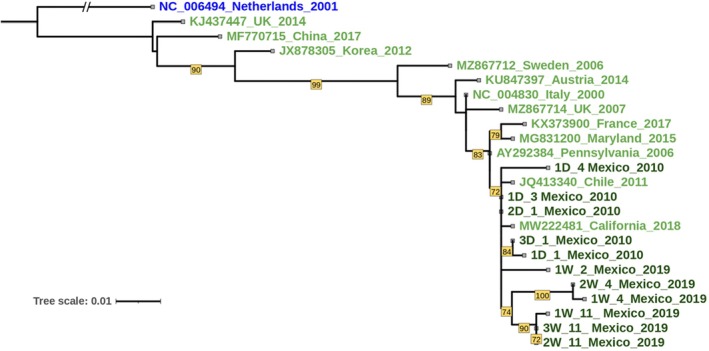
Phylogeny of DWV isolates from the Yucatan Peninsula, Mexico, based on 403 bases of the RdRp gene of 11 DWV‐A isolates from four geographical locations in the Yucatan Peninsula in 2010 and 2019 (dark green) as well as 12 isolates of DWV‐A from across the world (light green). DWV‐B from the Netherlands (blue) is used as outgroup to root the tree. Bootstrap (1000 replicates) support > 70% is shown. The scale bar shows the nucleotide substitution rate per site. For Mexican samples, all of which were generated in this study, each location code comprises a unique two‐digit code followed by an underscore and then a location number, as given in Tables S1 and S5).

DWV RdRp partial gene sequences from drone and worker AHBs collected in 2010 and 2019 were closely related to each other, with a similarity > 97% (Figure 2). Their relationship with 12 DWV‐A sequences from the NCBI database reflects geography; Yucatecan isolates are more similar to other American isolates and less similar to European and Asian isolates (Figure 2). Haplotype network analysis of DWV‐A sequences confirms the close relationship of Yucatecan sequences between 2010 and 2019 as well as with DWV‐A isolates from the USA, forming a relatively discrete cluster (Figure S4). Additional clusters in the network are composed of sequences from Europe and Asia in a pattern suggesting isolation by geographic distance (Figure S4).

### 3.3 Epidemiological Models Describing the Blocking of DWV‐B's Spread by DWV‐A

To explore why DWV‐B has not taken over from DWV‐A in the Yucatan Peninsula, we resort to our epidemiological model. Take a population of honey bees in which DWV‐A is well‐established and at a relatively high frequency, as is likely the case in the Yucatan Peninsula in 2010 or earlier. Assume DWV‐B enters this population at very low frequency (*b*_*0*_ = 0.01). Assuming that the transmission rate of DWV‐B is higher than that of DWV‐A, and using three different variants of the model whose basics are defined in Section 2.4, we now explore the parameter space under which DWV‐B could not displace DWV‐A. In this way, we aim to simulate the genotype dynamics of DWV in ABHs of the Yucatan Peninsula and potentially elsewhere where *A. mellifera* survives with little or no management of DWV‐vectoring varroa mites.

Here, we use the terms ‘normal pattern’ and ‘inverted pattern’ to refer to the scenarios in which DWV‐B can and cannot dominate pre‐established DWV‐A in a population of hosts, respectively. We call DWV‐B dominance normal as it is documented from Europe and the USA that this genotype has or is replacing DWV‐A (Paxton et al. 2022).

#### 3.3.1 Independent Spreading (Model 2)

We first assume DWV‐A and DWV‐B do not interact, i.e., the existence of one of them does not inhibit the transmission rate of another (*m*_*A*_ = *m*_*B*_ = 1), both viruses spread through the population independently. Under these conditions, Model 1 reduces to:

$$\text{Model}\;2:\left\{\begin{array}{c} \mathrm{da}/\mathrm{dt}=a\;\left(1-a\right)\;\mu_{A}-a\nu_{A}\\ \mathrm{db}/\mathrm{dt}=b\;\left(1-b\right)\;\mu_{B}-b\nu_{B} \end{array}\right.$$

These two differential equations can be solved analytically; the trajectory (the change in frequency over time) of each viral genotype X is then given by:

(1) $$x\left(t\right)=\frac{\Delta_{X}}{\left[\frac{\Delta_{X}}{x_{0}}-\mu_{X}\right]e^{-t\Delta_{X}}+\mu_{X}}$$

where $\Delta_{X}=\mu_{X}-\nu_{X}$ and $x_{0}$ is the initial frequency of viral genotype X.

We are interested in the equilibrium behavior of the system. Letting time approach infinity, the first term in the denominator approaches zero, which reduces Equation (1) to *x* (*t* = ∞) = 1‐*ν*_*X*_/*μ*_*X*_, which is the frequency of genotype X at equilibrium. Considering both genotypes, the whole system has four fixed (equilibrium) points, among which the last is stable:

$$\left[\begin{array}{c} \tilde{a}\\ \tilde{b} \end{array}\right]=\left[\begin{array}{c} 0\\ 0 \end{array}\right],\left[\begin{array}{c} 0\\ 1-\frac{\nu_{B}}{\mu_{B}} \end{array}\right],\left[\begin{array}{c} 1-\frac{\nu_{A}}{\mu_{A}}\\ 0 \end{array}\right],\left[\begin{array}{c} 1-\frac{\nu_{A}}{\mu_{A}}\\ 1-\frac{\nu_{B}}{\mu_{B}} \end{array}\right]$$

and where $\overset{`}{a}$ and $\overset{`}{b}$ are the frequencies of DWV‐A and DWV‐B at equilibrium. The first fixed point of Model 2 is trivial: no virus. The second and the third fixed points occur when only one virus exists in the population, for which the equilibrium virus frequency is given by 1‐ *ν*/*μ*; these are (locally) stable equilibria, stable only in the absence of the other viral genotype. The fourth point is the only stable state of the system (global stable equilibrium) when two genotypes of the virus coexist; regardless of their initial frequencies, the equilibrium values of their frequencies are given by the fourth fixed point shown above.

**FIGURE 3:**
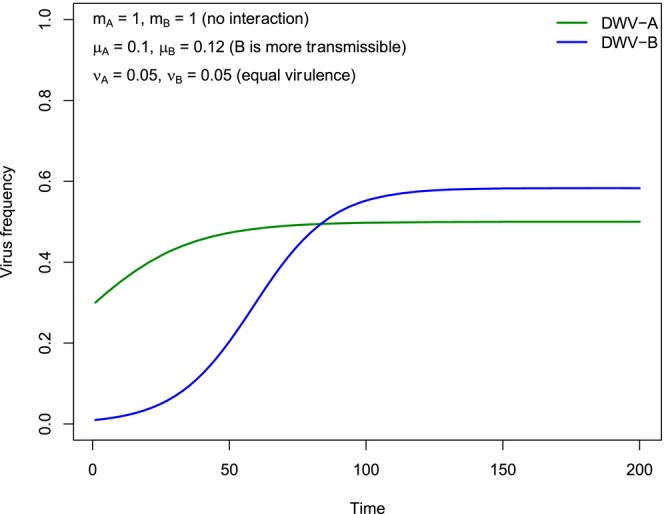
Dynamics of viral frequencies in the absence of a superinfection interaction (Model 2). Simulation of the frequency of two viral genotypes (using Equation 1) is shown when there is no interaction between genotypes and the transmission rate of DWV‐B is higher than that of DWV‐A, inspired by epidemiological data from the literature. With starting frequencies of 0.30 and 0.01 for DWV‐A and DWV‐B, respectively, both genotypes rise to high prevalence. The frequency of DWV‐B can increase with time even if it has a higher virulence than DWV‐A if the difference in virulence between genotypes does not exceed a threshold, which in this case is 0.06.

For initial frequencies of DWV‐A and DWV‐B of 0.30 and 0.01, respectively, Figure 3 shows the change in frequency of both genotypes of DWV with time, as predicted by Model 2 (no interaction between different genotypes), when the transmission rate of DWV‐B is higher than that of DWV‐A (*μ*_*B*_ = 0.12 vs. *μ*_*A*_ = 0.1) and the fatality rate (*ν*_*X*_) of both viral genotypes is the same. In the absence of any interaction between viral genotypes, the frequency of both genotypes increases until they attain an equilibrium value (Figure 3), which is reached regardless of initial frequencies (data not shown). The equilibrium values of the two genotypes are independent of each other and are only determined by the ratio of fatality rate to transmission rate (*ν*_*X*_/*μ*_*X*_). DWV‐B fails to reach a frequency higher than DWV‐A only if its fatality rate exceeds the threshold of (*μ*_*B*_ *ν*_*A*_)/*μ*_*A*_. We infer from the epidemiology of DWV‐B in Europe and the USA that the fatality rate of DWV‐B is not so high as to reduce its frequency in a population of hosts.

#### 3.3.2 Mutual Inhibition (Model 1)

Model 2 assumes no interaction between DWV‐A and DWV‐B. However, the pattern observed in the frequency of these two genotypes in the UK, Germany, and Italy (Kevill et al. 2021; Paxton et al. 2022) suggests some form of negative interaction between genotypes because an increase in the frequency of DWV‐B coincides with a decrease in the frequency of DWV‐A. Under the scenario of a population of hosts and two pathogen variants, the order of infection of a host may determine the probability that it becomes co‐infected; a pathogen variant that is already established in a host may inhibit infection by another variant (priority effect: Bashey 2015), which is a form of SIE. In order to capture this interaction, we set the mutual interaction terms in Model 1 to be nonzero.

Setting equal fatality rates for both viral genotypes for simplicity, we are interested in the conditions under which the equilibrium frequency of DWV‐A is higher than DWV‐B (even though the transmission rate of the latter is higher), i.e., $\overset{`}{a}>\overset{`}{b}$, which occurs when:

(2) $$m_{B}<\frac{1}{2\mu_{B}\left(\mu_{A}-\nu \right)}\left[\mu_{A}\;\left(\mu_{A}+\mu_{B}-2\nu \right)\;m_{A}+\left(\mu_{A}-\mu_{B}\right)\;\left(2\nu +\sqrt{\mu_{A}}\;\sqrt{\mu_{A}.m_{A}^{2}+4\nu -4\nu m_{A}}\right)\right]$$

where *m*_*B*_ indicates the extent to which the transmission rate of DWV‐B is inhibited by genotype A, and where a small value of *m*_*B*_ means strong inhibition.

**FIGURE 4:**
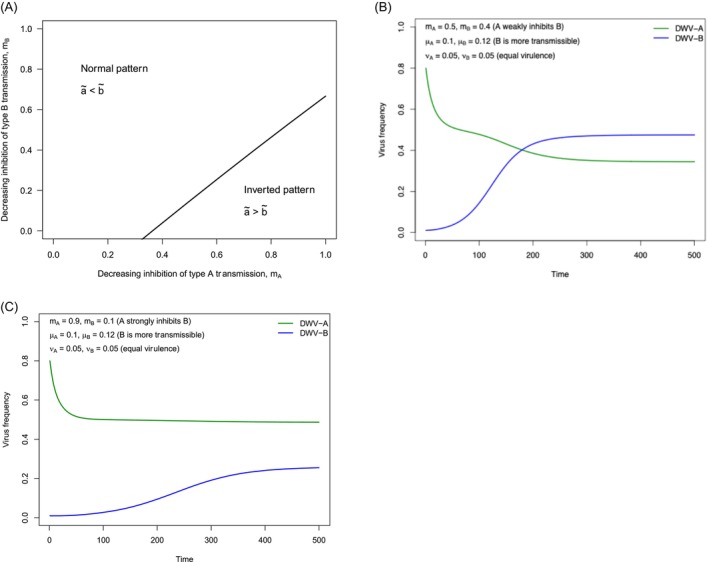
Equilibrium profile and dynamics of DWV‐genotype frequencies with superinfection exclusion (Model 1). (A) The parameter landscape from the basic model (Model 1) describing the epidemiology of the two genotypes of DWV, where two different patterns (normal pattern, B dominates A, versus inverted pattern, A dominates B) occur for different values of interaction (inhibition) between two viral genotypes: *M*_*A*_ and *m*_*B*_. DWV‐A can prevent the establishment of DWV‐B in only a small part of the parameter landscape because the transmission rate of DWV‐B is higher than that of DWV‐A. (B) When DWV‐A weakly inhibits DWV‐B, the latter dominates at equilibrium (‘normal pattern’). (C) When the inhibition of DWV‐B imposed by DWV‐A exceeds a threshold, given in Equation (2), DWV‐B cannot dominate in a host population with a high pre‐existing prevalence of DWV‐A (‘inverted pattern’).

When *m*_*B*_ drops below such a threshold, Model 1 reveals that genotype A has a strong enough effect to prevent DWV‐B establishment in a host already infected by DWV‐A. Figure 4A shows the corresponding parameter landscape described in Equation (2) where, for some parameter values of the interaction terms *m*_*A*_ and *m*_*B*_, DWV‐B (which has a higher transmission rate) can replace DWV‐A (‘normal pattern’ scenario) and for other values it cannot (‘inverted pattern’ scenario, DWV‐A limits DWV‐B). Figure 4B,C show the dynamics of the model under two conditions, one representing the ‘normal pattern’ scenario and another representing the ‘inverted pattern’ scenario. Here, we set the initial frequency of DWV‐A to 0.80, which more realistically reflects our observational data from the Yucatan Peninsula in 2010. However, only under a limited range of parameter values can DWV‐A maintain its high frequency and limit the spread of DWV‐B (Figure 4A).

Note, though, that mutual inhibition nevertheless leads to maintenance of both genotypes within the population of hosts (Figure 4B,C) and not the exclusion of one genotype by the other, though exclusion seems to occur in the UK, Germany, and Italy (Kevill et al. 2021; Paxton et al. 2022).

#### 3.3.3 Recombination Meltdown (Model 3)

We now hypothesize a scenario in which a new immigrant virus (DWV‐B) enters an already‐established population of DWV‐A and, whenever these two viral genotypes mix in the same host, they can undergo a recombination process to produce a recombinant variant with a high fatality rate. As a result, DWV‐B will be quickly removed from the population, leaving DWV‐A at a high frequency despite the fact that DWV‐B has a higher transmission rate compared to DWV‐A. This represents a strong form of SIE.

Model 3 describes these dynamics, incorporating a high recombination rate when two genotypes of DWV coexist. In this model, R (with transmission rate of $\mu_{R}$, fatality rate of $\nu_{R}$, and a frequency of *r*) indicates a recombinant. We assume for simplicity that there is only one type of DWV recombinant. The change in viral genotypes (A, B, and R) is then given by:

$$\text{Model}\;3:\left\{\begin{array}{c} \mathrm{da}/\mathrm{dt}=\left(1-a\right).a.\mu_{A}-a.b.\left(1-r\right).\alpha -a.\nu_{A}\\ \mathrm{db}/\mathrm{dt}=\left(1-b\right).b.\mu_{B}-a.b.\left(1-r\right).\alpha -b.\nu_{B}\\ \mathrm{dr}/\mathrm{dt}=\left(1-r\right).r.\mu_{R}+a.b.\left(1-r\right).\alpha -r.\nu_{R} \end{array}\right.$$

where α indicates the recombination rate of viral genotypes in a co‐infected host.

**FIGURE 5:**
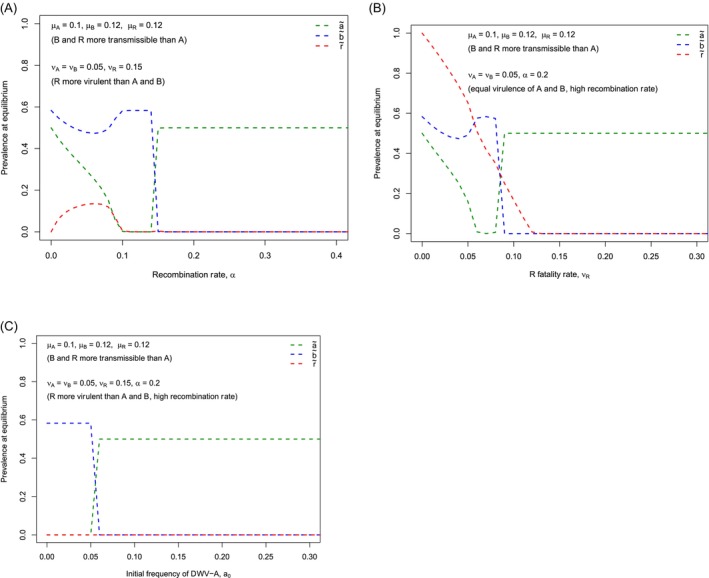
Equilibrium profile and dynamics of DWV‐genotype frequencies with SIE through recombination meltdown (Model 3). Here we investigate a honey bee host population with a pre‐established high prevalence of DWV‐A and low frequency of new immigrants of DWV‐B, in which the two viral genotypes, A and B, undergo recombination events at a rate of *α* and when they coexist and generate a recombinant virus (denoted by R), which has its own transmission rate (*μ*_*R*_) and fatality rate (*ν*_*R*_). The equilibrium frequencies of viral genotypes A, B, and R ($\overset{`}{a}$, $\overset{`}{b}$, and $\overset{`}{r}$, respectively) are shown as a function of recombination rate (A), recombinant fatality rate (B), and initial frequency of DWV‐A (C). In such a population, when the recombination rate, recombinant fatality rate, and initial frequency of DWV‐A are high enough, genotype B is completely excluded by DWV‐A, despite the fact that DWV‐B has a higher transmission rate compared with DWV‐A. Releasing any of these three conditions can result in the complete replacement of DWV‐A by DWV‐B.

In this model, different genotypes of DWV do not influence each other's transmission rates, i.e., *m*_*A*_ = *m*_*B*_ = 1. When *α* = 0 (no recombination event) and *r* = 0 (no recombinant), this model reduces to model 1. Since the frequency of each genotype depends on the frequency of other genotypes, this model cannot be solved analytically. However, we can inspect the equilibrium profile numerically. Figure 5 shows the equilibrium values of three genotypes of the virus (DWV‐A, DWV‐B, and recombinant) under different values of the recombination rate (Figure 5A), recombinant fatality rate (Figure 5B), and the initial frequency of genotype A (Figure 5C). When the recombination rate, the recombinant fatality rate, or the initial frequency of genotype A is sufficiently high, DWV‐B cannot enter and spread to high frequency in a host population already infected by DWV‐A as a consequence of recombination meltdown. Hence, the ‘inverted pattern’ is observed: DWV‐A maintains a high frequency in the population of hosts, DWV‐B cannot invade and is suppressed to extremely low prevalence. Parameter values for DWV‐A to maintain its dominance over DWV‐B are broader in Model 3 (recombination meltdown; Figure 5) than for Model 1 (mutual inhibition; Figure 4), yet releasing any of the three conditions permitting genotype A to dominate leads to replacement by DWV‐B and the ‘normal pattern’ (Figure 5).

## 4 Discussion

We here reveal the presence of DWV‐A and DWV‐B in AHBs of the Yucatan Peninsula of Mexico since 2010. However, contrary to our original hypothesis, DWV‐B has not replaced DWV‐A and has remained at very low prevalence up to 2019, whereas DWV‐A has maintained its predominance. Our epidemiological modeling suggests that this ‘inverted pattern’ of apparent dominance of DWV‐A over DWV‐B in tropical Yucatan may be the consequence of a strong form of SIE. One potential form of strong SIE is inter‐genotype recombination meltdown, for which we develop an epidemiological model that informs on the parameter space within which recombination could lead to blocking of one viral variant by another.

### 4.1 DWV‐A Dominance in Yucatecan AHBs

Varroa and associated viruses likely entered the Yucatan Peninsula from North America (Traynor et al. 2020), possibly with imported honey bees. This view is supported by our phylogeny of DWV‐A isolates of the Yucatan Peninsula, which are located close to US isolates. It is therefore likely that DWV‐A was first brought to the Yucatan Peninsula on or before 1994 with varroa mites from the USA (Medina and Martin 1999; Traynor et al. 2020), and has subsequently risen to high prevalence in the region's honey bees. Other studies (Guzmán‐Novoa et al. 2016; Correa‐Benítez et al. 2023) also support the view that DWV is widespread in Mexico's AHBs, though these studies used generic primers unable to differentiate DWV genotype. Considering the major role that the varroa mite plays as a vector of DWV, the expansion of varroa to encompass South America (Paraguay since 1971) and North America (USA since 1987) (Rosenkranz et al. 2010; Traynor et al. 2020), including the Yucatan Peninsula (since 1994; Medina and Martin 1999), has likely been accompanied by the spread of RNA viruses, including DWV (Martin et al. 2012; Mondet et al. 2014; Hasegawa et al. 2023; Doublet et al. 2024).

Data from continental North America as well as from Hawaiian islands reveal a rise in the prevalence of DWV‐B (Ryabov et al. 2017; Grindrod et al. 2021; Zhang et al. 2024), where DWV‐A may be in a process of replacement by DWV‐B, as already seen in European countries (Kevill et al. 2021; Paxton et al. 2022). Though DWV‐A remained the predominant genotype in the USA in 2016 (Ryabov et al. 2017), European data as well as epidemiological modeling have predicted an increase in DWV‐B's prevalence in North America and a decline or loss of DWV‐A (Paxton et al. 2022), a view which recent data support (Hesketh‐Best et al. 2024, cf. Lamas et al. 2025).

DWV‐B was first described from honey bees and varroa mites collected in 2001 in the Netherlands (Ongus et al. 2004). DWV‐B's first detection in the Americas is from 2010, when it was found in only two of 71 US colonies (Ryabov et al. 2017), suggesting that DWV‐B is a relatively recent (2010) arrival to continental America. DWV‐B's entry to the Yucatan Peninsula is unknown, though possibly also associated with the importation of honey bees (queens or package bees) and associated varroa mites from the USA. That DWV‐B was first detected in 2010 not only in the USA (Ryabov et al. 2017) but also in Canada (Doublet et al. 2024) and in the Yucatan Peninsula (this study), in all cases at very low prevalence compared to DWV‐A, may reflect a recent, common introduction to North America. Phylogeographic analysis of sequence data for these and other DWV‐B isolates would help resolve the question of a single versus multiple introductions to North America and their origin, as recently undertaken for DWV‐A (Hasegawa et al. 2023).

Here we show that DWV‐B was already present in honey bees of the Yucatan Peninsula in 2010. Yet our empirical dataset reveals no change in the absolute prevalence of DWV‐B in 9 years, with no evidence of DWV‐B replacing DWV‐A, unlike the situation in temperate regions of North America and Europe (Ryabov et al. 2017; Kevill et al. 2021; Paxton et al. 2022).

Though we saw a drop in the absolute prevalence of DWV‐A in Yucatecan honey bees between 2010 and 2019, we hypothesize that this is a consequence of the different colony members that we analyzed: drones collected at DCAs in 2010 versus workers collected at flowers in 2019. Differences in absolute viral prevalence between drones and workers likely arise because drone pupae are preferred as hosts over worker pupae by varroa mites (Rosenkranz et al. 2010). Drones collected at DCAs therefore often have a high prevalence of viruses, including DWV (Yáñez et al. 2012; Forfert et al. 2016; Amiri et al. 2016), possibly higher than the prevalence in workers collected at flowers (our 2019 dataset). However, our results should be treated with caution because, by sampling different colony members in 2010 and 2019, our data may not accurately reflect the evolutionary epidemiology of DWV in the Yucatan Peninsula. Renewed sampling of honey bee drones at DCAs in the Yucatan would be needed to test whether DWV‐A has indeed dropped in absolute prevalence, and whether DWV‐B has been blocked in its expansion.

The status of DWV‐B in the honey bees of other Latin American countries nevertheless seems to reflect the picture in Yucatan. DWV‐A seems to be dominant over DWV‐B in Argentina and Chile, possibly because of DWV‐B's recent detection in those countries in 2016 and 2015, respectively (Brasesco et al. 2020; Riveros et al. 2020). A recent study of DWV genotypes in Argentinian honey bees collected in 2018/2019 revealed only DWV‐A (at a prevalence of up to 82%) and a lack of DWV‐B (Gonzalez et al. 2024), suggesting little or no expansion of DWV‐B in that country. Even after 9 years of presence in tropical SE Mexico, we found that DWV‐B has remained at very low prevalence and DWV‐A continues to be the dominant DWV variant.

Our phylogenetic analyses corroborate the presence of the same DWV‐A variant in honey bees from the Yucatan Peninsula at both of our sampling time points, 2010 and 2019, suggesting long‐term stability of DWV‐A in Yucatecan honey bees. The broad host range of DWV‐A and its low apparent virulence in arthropod communities suggest that DWV is a generalist virus of many host species (Martin and Brettell 2019), which could explain why DWV‐A remains dominant and without marked genetic change in a decade. The eco‐evolutionary relevance of this suggestion is that DWV may spill over and infect other insects, including native bee species such as the culturally emblematic stingless bee *Melipona beecheii* Bennett, 1831 of the Yucatan Peninsula (Fleites‐Ayil et al. 2023).

### 4.2 Modelling Suggests a Strong Form of SIE Is Needed to Block DWV‐B's Rise in Prevalence

Paxton et al.'s (2022) epidemiological ‘compartment’ model accounted for the spread of DWV‐B in honey bee populations already infected by DWV‐A in temperate regions of the world; it mirrors our current Model 2. It also demonstrated that, in the absence of inhibition, the frequency of each viral genotype changes with time independently until each reaches equilibrium, i.e., both genotypes reach and remain at high prevalence in a population of honey bees. However, the observed patterns of DWV‐genotype dynamics in Europe (Kevill et al. 2021; Paxton et al. 2022) and the USA (Ryabov et al. 2017; Grindrod et al. 2021) suggest a form of negative interaction (SIE) between these two variants because the prevalence of DWV‐A decreases to quasi‐zero as the prevalence of DWV‐B increases. Based on this observation, Paxton et al. (2022) introduced mutual inhibition between viral genotypes to explain patterns of genotype prevalence, mirroring our current Model 1 (weak SIE); modeling suggests replacement of DWV‐A by DWV‐B, though over many generations (50–250 model iterations, equivalent to 12–60 years), as seems to be occurring in Europe and the USA.

Our empirical data on DWV‐genotype dynamics in Yucatan, in contrast, suggest that DWV‐A blocks the spread of DWV‐B. Since the transmission rate of DWV‐B is assumed to be higher than that of DWV‐A, weak inhibition of DWV‐B by DWV‐A cannot theoretically block the spread of DWV‐B. However, strong inhibition by DWV‐A could suppress the spread of DWV‐B despite the latter's higher rate of transmission, which results in what we refer to as the ‘inverted pattern’.

Mordecai et al. (2016) hypothesized two plausible mechanisms of SIE between DWV‐A and DWV‐B: (i) the resources of the host (e.g., host cell membrane binding sites, intracellular sites for RNA replication) are already consumed or occupied by the first pathogen and (ii) host immunity is already triggered by the first pathogen, which makes it hard for the second pathogen to establish. These possibilities might explain priority effects, which are widespread in host‐multiparasite systems (Bashey 2015) and have been observed during co‐infection by two or more viral variants (Jokinen et al. 2023), including by DWV infecting honey bees (Gusachenko et al. 2021). Our modeling (Model 1) of DWV‐genotype dynamics, in which DWV‐A is already established in a honey bee population (our Equation 2), demonstrates that genotype B may not be able to establish in the host population if DWV‐A is at high enough initial frequency, possibly through either or both of Mordecai et al.'s (2016) two proposed mechanisms of SIE. Yet they do not easily account for the complete replacement of one variant by the other, which has apparently happened in the UK, Germany, and Italy in DWV‐B's near‐complete exclusion of DWV‐A (Kevill et al. 2021; Paxton et al. 2022), and which may be currently holding DWV‐B to quasi‐zero prevalence in the Yucatan Peninsula.

Inspired by viral genotype dynamics in Yucatecan honey bees and following a third suggestion for SIE by Mordecai et al. (2016) in which DWV‐A might have been ‘recombined out’ by DWV‐B in a British population of honey bees, we developed Model 3, incorporating recombination meltdown as a strong form of SIE. Model 3 suggests that DWV‐A may prevent the establishment of DWV‐B in the Yucatan Peninsula through a strong form of SIE brought on by recombination between genotypes when DWV‐B first enters a host population. It highlights three criteria that need to be fulfilled for DWV‐A to block the spread of DWV‐B: (i) a high initial frequency of DWV‐A; (ii) a high recombination rate on superinfection; and (iii) a high recombinant fatality rate. Our empirical data suggest criterion (i) is fulfilled: DWV‐A is at high prevalence in the Yucatan Peninsula.

Whether criteria (ii) and (iii) are fulfilled is an open question, though evidence suggests that they might also hold in this system. Viruses can exhibit high rates of recombination, especially so (+)ssRNA viruses, though there is little evidence that selection has acted on the rate of recombination, and recombination itself is viewed as a mechanistic byproduct of RNA polymerase processivity (Holmes 2009; Simon‐Loriere and Holmes 2011). Diverse recombinants between DWV‐A and DWV‐B have been reported within honey bee populations of the USA and Europe using next generation sequencing (NGS) data (Hesketh‐Best et al. 2024; Sircoulomb et al. 2025), suggesting a significant rate of inter‐genotype recombination in DWV, supporting the second criterion that promotes recombination meltdown. Though homologous recombination between two viral variants could theoretically lead to a positive epistatic interaction, conferring a selective advantage on a chimeric A/B viral genotype, it could also lead to negative epistasis. Indeed, viral recombination is typically thought to produce deleterious genotypes that are removed by purifying selection (Posada et al. 2002; Simon‐Loriere and Holmes 2011), as inferred in HIV‐1 recombinants of the envelope gene (Simon‐Loriere et al. 2009). Moreover, since DWV is an RNA virus, the correct spatial structure of the genomic molecule is necessary for viral replication (Holmes 2009). As such, many viral recombinants are likely dysfunctional. The consequence of this process may then be recombination meltdown, in which two viral RNA molecules (one of each variant) recombine when co‐infecting the same host cell and perish. These views provide support for the third criterion: a high recombination fatality rate of recombinants, which is equivalent to error catastrophe in the quasispecies theory of viruses (Lauring and Andino 2010).

Under this scenario, we hypothesize that a recombination process could eventually lead to suppression of DWV‐B in the Yucatan Peninsula despite DWV‐B's higher fitness if it enters as a rare variant into the host population already harboring DWV‐A at high prevalence. Our modeling suggests that recombination meltdown allows considerable parameter space in which DWV‐A can block DWV‐B's spread, despite DWV‐B's greater transmissibility.

Of course, the mere existence of A/B recombinant genotypes of DWV, as recently detected in the USA and Europe (Hesketh‐Best et al. 2024; Sircoulomb et al. 2025), suggests that not all recombinants are dysfunctional; i.e., that recombination meltdown might not be the mechanism by which DWV‐A blocks the spread of DWV‐B in the Yucatan Peninsula. In this case, our Model 3 might nevertheless allow predictions over the evolutionary epidemiology of DWV to be made. A challenge will be to parameterize Model 3 with data on the relative fitness of different recombinants because the field lacks a DWV cell culture system to allow efficient assaying of the comparative fitness of different viral genotypes.

We parsimoniously note that mutual inhibition (*m*_*A*_ = *m*_*B*_) through recombination meltdown (or another mechanism of SIE) might better explain why DWV‐B has so rapidly replaced DWV‐A in many temperate regions of the world, where DWV‐A's prevalence was initially lower than in the Yucatan Peninsula and where DWV‐B has entered and rapidly risen to high prevalence whilst DWV‐A has been eliminated. An explanation for the rapid replacement of DWV‐A by DWV‐B in temperate regions might relate to the region's marked seasonality. *Apis mellifera* colonies may enter winter with high varroa mite counts, high viral prevalence, and high viral burden (titer) per individual bee, but varroa mites and infected bees likely suffer high overwinter mortality; therefore, viral prevalence and burden are much lower in spring (Natsopoulou et al. 2017; Wham et al. 2024; Molinatto et al. 2025). In essence, a winter break in worker brood production may knock down DWV prevalence, allowing DWV‐B to spread more rapidly through an uninfected host population than in a warm or tropical region where *A. mellifera* brood production, varroa counts, and viral prevalence remain high throughout the year. We note that assiduous beekeeping management to reduce varroa counts per colony and lower viral prevalence among worker bees within the colony may ironically help DWV‐B spread through a host population.

It is possible that the negative interaction brought on among viral variants following superinfection could be influenced by other factors that, when combined, benefit the establishment or dominance of one viral variant over another, i.e., DWV‐A in the tropical Yucatan Peninsula. For example, a warm, subtropical environment such as that of SE Mexico may lower the susceptibility of honey bees to viral infection (Anguiano‐Baez et al. 2016), with consequences for viral epidemiology, namely greater difficulty for a virus to become established in a host population. Alternatively, or in addition, the genetic origin of the honey bees could play an important role in host susceptibility to viral infection. It is well documented that AHBs show resistance or tolerance to numerous pathogens, including *V. destructor* (Guzmán‐Novoa et al. 1999, 2024; Martin and Medina 2004) and viral infections (Hamiduzzaman et al. 2015), compared with European *A. mellifera* . Also, differences in susceptibility to viruses have been recorded among other tropical honey bee species co‐occurring with European *A. mellifera* ; in North Thailand, imported European *A. mellifera* has higher viral prevalence compared with other sympatric and native honey bee species (Chantaphanwattana et al. 2023). Possible differences in viral tolerance among honey bee species and hybrids (AHB) could also help explain why DWV‐A continues its dominance over DWV‐B in the Yucatan Peninsula. That honey bees in South Africa have been reported to harbor DWV‐B at high prevalence (Strauss et al. 2013) suggests that AHBs, which are of sub‐Sahara African ancestry, may not differ in tolerance to DWV variants from those of European ancestry or that (lack of) seasonality disfavors DWV‐B's expansion. Honey bee genetic background may nevertheless impact viral epidemiology and demands closer investigation.

## 5 Conclusion

That DWV‐B's expansion is underway in the Americas is an interesting idea that should be tested in the coming decades. Evidence for the strong negative impact of DWV‐B on honey bee populations in Europe (e.g., Natsopoulou et al. 2017) as well as its presence in different host species (Fürst et al. 2014; de Souza et al. 2019; Martin and Brettell 2019; Manley et al. 2019; Maurer et al. 2024) leads to the suggestion that DWV‐B may be in an expansion process in the Americas and other world regions, with potential deleterious impacts on infected hosts. Though our evidence suggests that DWV‐B may be blocked from expansion in the Yucatan Peninsula and potentially elsewhere in Latin America, this may be a temporary phenomenon reflecting a local equilibrium; given DWV‐B's apparently higher transmission than that of DWV‐A, the long‐term, global equilibrium is likely the ‘normal pattern’ of DWV‐B dominance and elimination of DWV‐A. DWV‐B should be considered an emerging threat for honey bee populations as well as for other bee species and pollinator diversity, including in the Neotropics (Fleites‐Ayil et al. 2023).

Superinfection is likely widespread among parasites (Rigaud et al. 2010; Schmid‐Hempel 2011), and is an inevitability during the process of replacement of DWV‐A by DWV‐B. It also has theoretical consequences for the evolution of virulence (Read and Taylor 2001; Alizon et al. 2013; Makau et al. 2022). The identification of diverse DWV‐A/B recombinants using NGS technologies (USA: Hesketh‐Best et al. 2024; Europe: Sircoulomb et al. 2025) suggests that recombination may be a regular occurrence in host honey bee populations infected by both DWV genotypes. A small number of surviving recombinants with a higher fatality rate represent a high risk for honey bees (e.g., Ryabov et al. 2014), one that deserves greater empirical and epidemiological scrutiny for the benefit of beekeeping with *A. mellifera* and wider invertebrate conservation.

## Ethics Statement

Research described herein did not require ethical approval and adheres to national and international rules.

## Conflicts of Interest

The authors declare no conflicts of interest.

## Supporting information


**Table S1:** Sampling information (location codes) at which AHB drones (2010) and AHB workers (2019) were sampled from the Yucatan Peninsula of Mexico and screened for DWV using generic (amplifying both genotypes A and B) and DWV‐genotype‐specific primers.
**Table S2:** qPCR primers used to amplify DWV and two host honey bee reference genes, β‐actin and RP49.
**Table S3:** qPCR data of Yucatecan AHB drones collected from four DCAs in 2010 and amplified using DWV‐generic primers.
**Table S4:** qPCR data of a subset of Yucatecan AHB drones collected from four DCAs in 2010 and amplified using DWV‐genotype‐specific primers.
**Table S5:** NCBI Accession Numbers for partial RdRp gene sequences of DWV‐A derived from Yucatecan AHBs: drones collected from DCAs in 2010 (*n* = 5 sequences) and workers collected from flowers in 2019 (*n* = 6 sequences).

**Figure S1:** DWV prevalence in AHB drones. DWV prevalence based on DWV‐generic primers (Table S2) in 250 AHB drones collected from DCAs in 2010, mapped by location in the Yucatan Peninsula of Mexico. Sample sites: 1. Dzoncahuich, 2. Cenotillo, 3. Izamal and 4. Espita. DWV prevalence at each location: Dzoncahuich 0.94 (60 of 64 drones), Cenotillo 0.83 (55 of 66 drones), Izamal 0.82 (37 of 45 drones); Espita 0.17 (13 of 75 drones); the prevalence of DWV averaged over all sites is 0.66.
**Figure S2:** DWV intensity of infection in AHBs from the Yucatan Peninsula, Mexico. DWV load as Cq value from DWV‐positive drones and workers sampled from the Yucatan Peninsula in 2010 and 2019, respectively. Box and whiskers plots show the median (dark bar), interquartiles (shaded), and 1.5 × interquartiles (whiskers); dots show original data points (mean Cq per individual). DWV‐A and DWV‐B are represented by green and blue shading, respectively. The 2019 data are from Fleites‐Ayil et al. (2023). The Cq value is inverse to the intensity of infection (viral titer or load).
**Figure S3:**. DWV‐A and DWV‐B prevalence (absolute and relative) in Yucatan, Mexico. (A) Absolute (including uninfected hosts) and (B) relative (excluding uninfected hosts) prevalence of DWV‐A and DWV‐B in AHBs in the Yucatan Peninsula of Mexico. DWV‐A dropped in absolute prevalence in workers collected in 2019 (*N* = 114) in comparison to drones collected in 2010 (*N* = 89) (Fisher exact test, *p* < 0.0001), while DWV‐B absolute prevalence across the same time frame remained low and did not change (Fisher exact test, *p* = 0.5944). The 2019 data are from Fleites‐Ayil et al. (2023).
**Figure S4:**. Median‐joining haplotype network of the 11 DWV‐A RdRp sequences (403 bases) from this study as well as 12 DWV‐A RdRp sequences from across the world (downloaded from NCBI). Identical sequences from the same host individual were pruned. Code names of samples comprise three parts: the 1st part denotes the individual code/GenBank Accession Number, the 2nd part denotes the country of origin, and the 3rd part denotes the date of sample collection (as in Figure 2). For Mexico samples, all of which were generated in this study, each location code comprises a unique two‐digit code followed by an underscore and then the location in the Yucatan Peninsula, as given in Table S1. Sequences (dots) are also colored based on location: red for Yucatan, Mexico (this study), green for elsewhere in N and S America, purple for Europe, and yellow for East Asia. Black dots represent putative, unsampled haplotypes, while a bar represents 1 base difference. GenBank Accession codes for the 11 Mexican sequences generated in this study are given in Table S5. Haplotype network constructed and visualized using PopART (https://popart.maths.otago.ac.nz/) (Leigh and Bryant 2015).

## Data Availability

Raw data underpinning this study are provided in the Supporting Information and downloadable at (zenodo web link will be provided on acceptance; DOI: https://doi.org/10.5281/zenodo.16583801); Excel file of data (Tables S1–S5) is uploaded for reviewers.
