# Supplementary material for: Superinfection Exclusion in Neotropical Honey Bees May Block DWV‐B, an Emerging Infectious Disease Variant of Deformed Wing Virus
Source: Evol Appl. 2025 Aug 12;18(8):e70143. doi: 10.1111/eva.70143 (PMC12340715; doi:10.1111/eva.70143)
Supplement: Supplementary file 2 — Figure S1: DWV prevalence in AHB drones. DWV prevalence based on DWV‐generic primers (Table S2) in 250 AHB drones collected from DCAs in 2010, mapped by location in the Yucatan Peninsula of Mexico. Sample sites: 1. Dzoncahuich, 2. Cenotillo, 3. Izamal and 4. Espita. DWV prevalence at each location: Dzoncahuich 0.94 (60 of 64 drones), Cenotillo 0.83 (55 of 66 drones), Izamal 0.82 (37 of 45 drones); Espita 0.17 (13 of 75 drones); the prevalence of DWV averaged over all sites is 0.66. Figure S2: DWV intensity of infection in AHBs from the Yucatan Peninsula, Mexico. DWV load as Cq value from DWV‐positive drones and workers sampled from the Yucatan Peninsula in 2010 and 2019, respectively. Box and whiskers plots show the median (dark bar), interquartiles (shaded), and 1.5 × interquartiles (whiskers); dots show original data points (mean Cq per individual). DWV‐A and DWV‐B are represented by green and blue shading, respectively. The 2019 data are from Fleites‐Ayil et al. (2023). The Cq value is inverse to the intensity of infection (viral titer or load). Figure S3:. DWV‐A and DWV‐B prevalence (absolute and relative) in Yucatan, Mexico. (A) Absolute (including uninfected hosts) and (B) relative (excluding uninfected hosts) prevalence of DWV‐A and DWV‐B in AHBs in the Yucatan Peninsula of Mexico. DWV‐A dropped in absolute prevalence in workers collected in 2019 (N = 114) in comparison to drones collected in 2010 (N = 89) (Fisher exact test, p < 0.0001), while DWV‐B absolute prevalence across the same time frame remained low and did not change (Fisher exact test, p = 0.5944). The 2019 data are from Fleites‐Ayil et al. (2023). Figure S4:. Median‐joining haplotype network of the 11 DWV‐A RdRp sequences (403 bases) from this study as well as 12 DWV‐A RdRp sequences from across the world (downloaded from NCBI). Identical sequences from the same host individual were pruned. Code names of samples comprise three parts: the 1st part denotes the individual code/GenBank Accession Numb [file EVA-18-e70143-s002.docx]

**Appendix**

**Supplementary Figure S1**


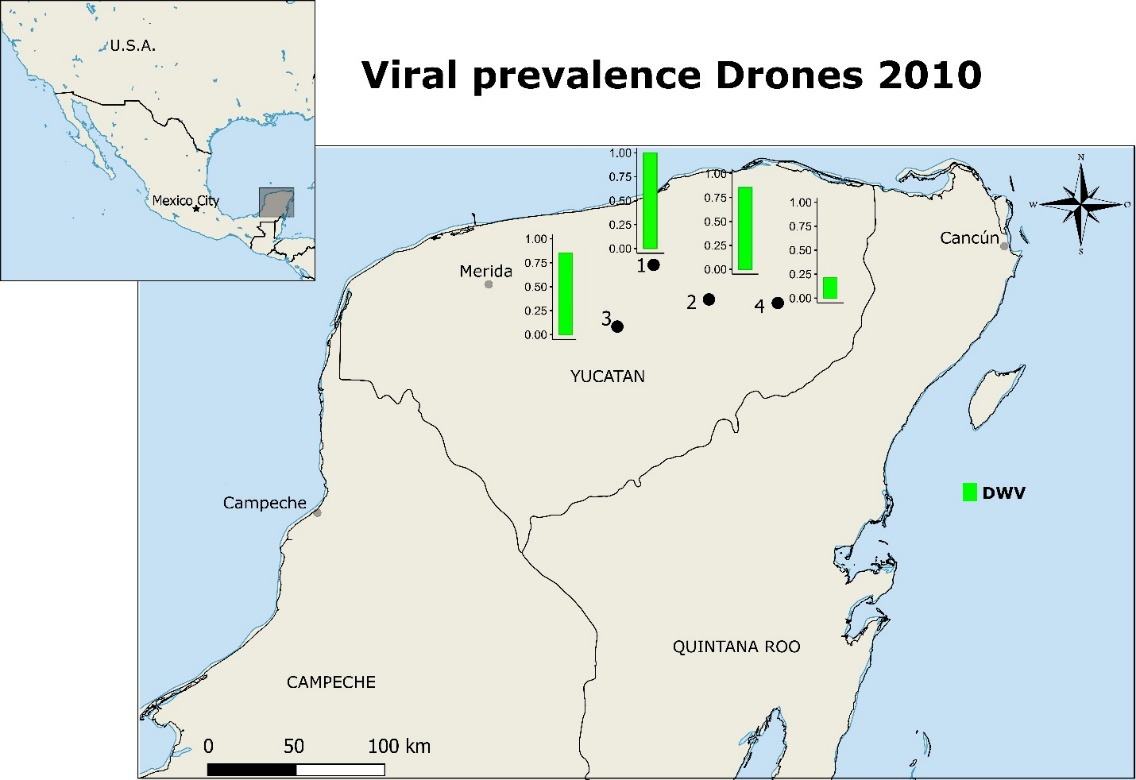


**Supplementary Figure S1**. **DWV prevalence in AHB drones.** DWV prevalence based on DWV-generic primers (Table S2) in 250 AHB drones collected from DCAs in 2010, mapped by location in the Yucatan Peninsula of Mexico. Sample sites: 1. Dzoncahuich, 2. Cenotillo, 3. Izamal and 4. Espita. DWV prevalence at each location: Dzoncahuich 0.94 (60 of 64 drones), Cenotillo 0.83 (55 of 66 drones), Izamal 0.82 (37 of 45 drones); Espita 0.17 (13 of 75 drones); the prevalence of DWV averaged over all sites is 0.66.

**Supplementary Figure S2**


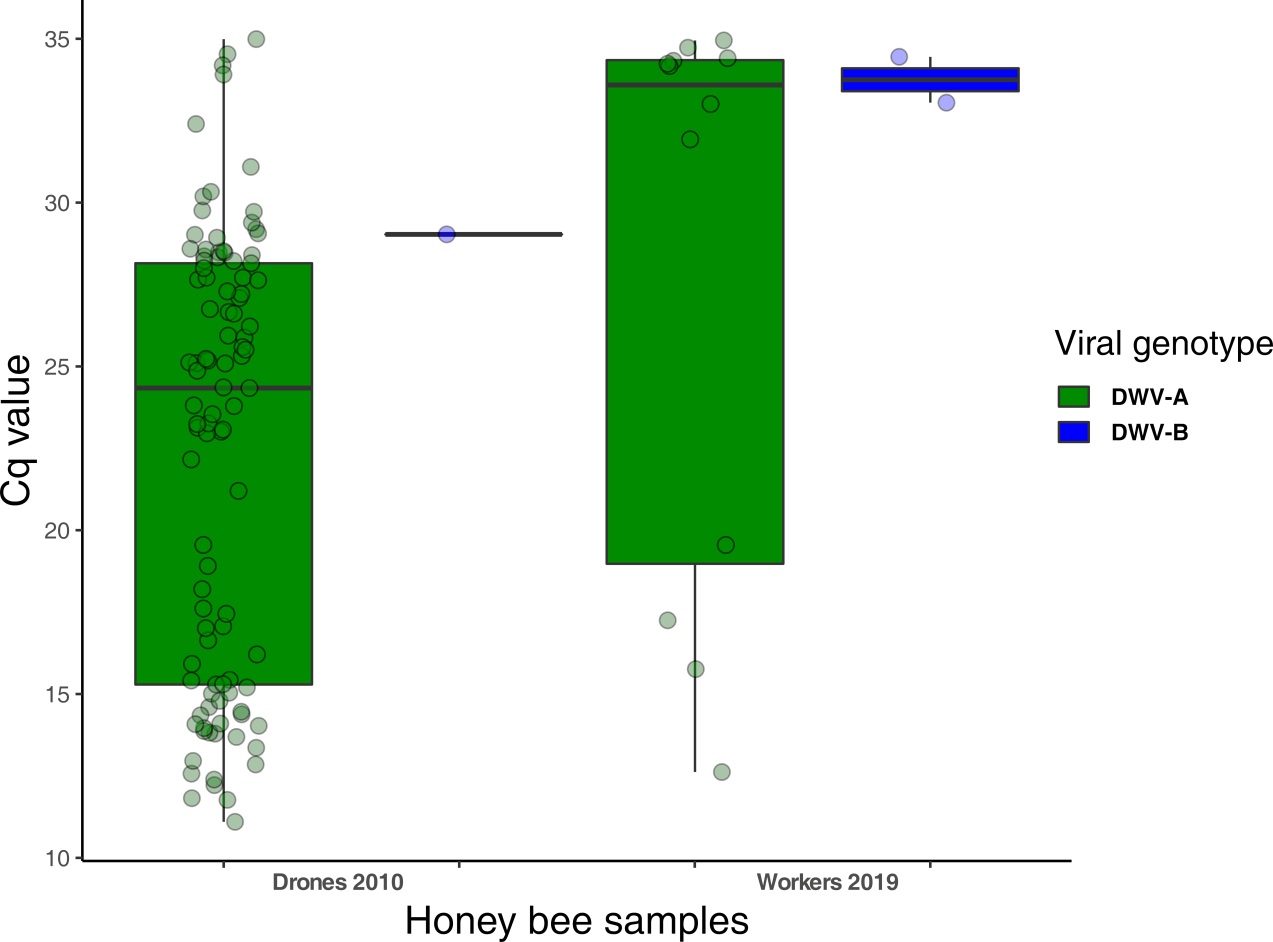


**Supplementary Figure S2. DWV intensity of infection in AHBs from the Yucatan Peninsula, Mexico.** DWV load as Cq value from DWV-positive drones and workers sampled from the Yucatan Peninsula in 2010 and 2019, respectively. Box and whiskers plots show the median (dark bar), interquartiles (shaded) and 1.5 x interquartiles (whiskers); dots show original data points (mean Cq per individual). DWV-A and DWV-B are represented by green and blue shading, respectively. The 2019 data are from Fleites-Ayil et al. (2023). The Cq value is inverse to the intensity of infection (viral titre or load).


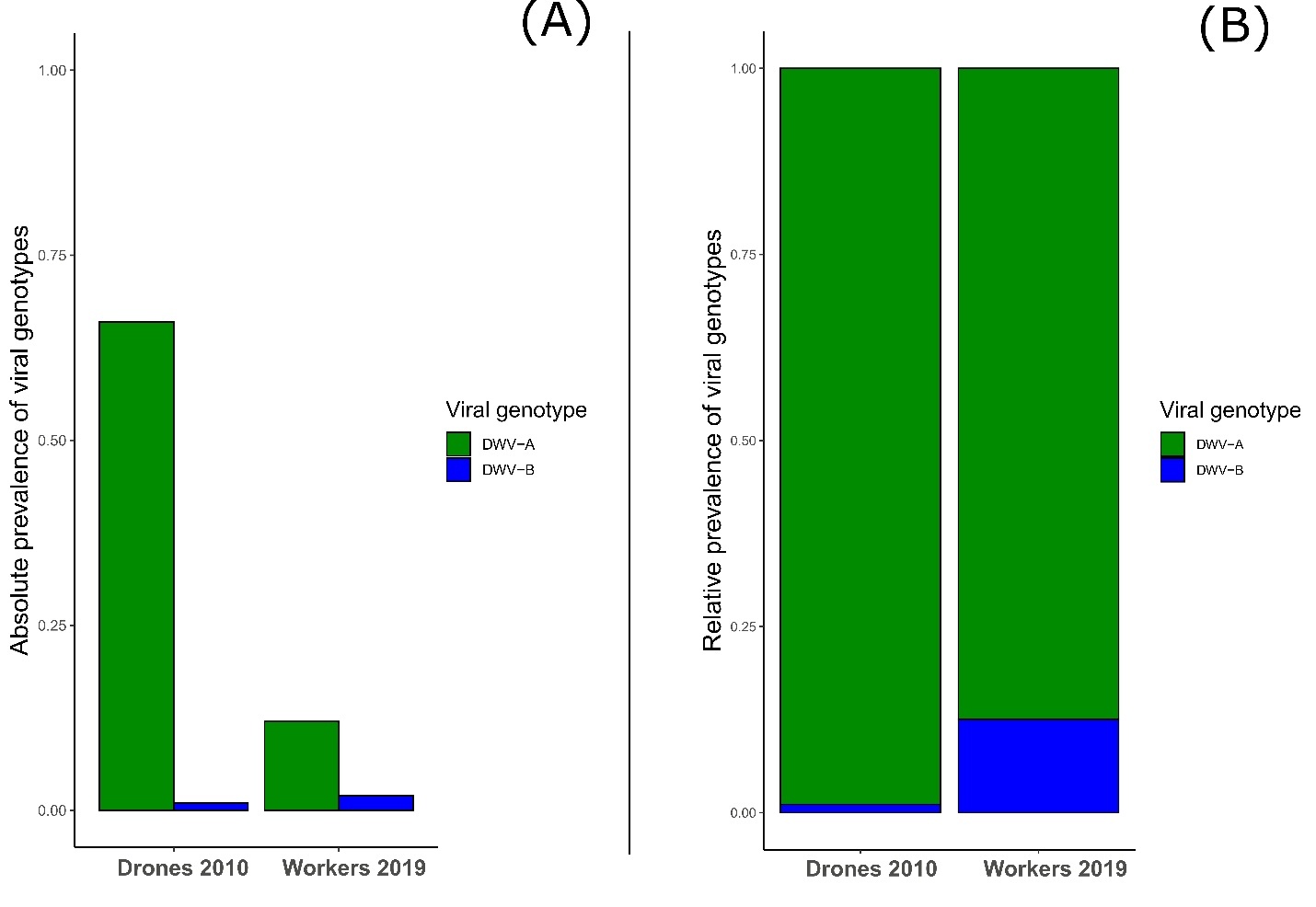
**Supplementary Figure S3**

**Supplementary Figure S3**. **DWV-A and DWV-B prevalence (absolute and relative) in Yucatan, Mexico**. **(A)** Absolute (including uninfected hosts) and **(B)** relative (excluding uninfected hosts) prevalence of DWV-A and DWV-B in AHBs in the Yucatan Peninsula of Mexico. DWV-A dropped in absolute prevalence in workers collected in 2019 (N = 114) in comparison to drones collected in 2010 (N = 89) (Fisher exact test, *P*<0.0001) whilst DWV-B absolute prevalence across the same time frame remained low and did not change (Fisher exact test, *P*=0.5944). The 2019 data are from Fleites-Ayil et al. (2023).

S**upplementary Figure S4**

**Supplementary Figure S4**. Median joining haplotype network of the 11 DWV-A RdRp sequences (403 bases) from this study as well as 12 DWV-A RdRp sequences from across the world (downloaded from NCBI). Identical sequences from the same host individual were pruned. Code names of samples comprise three parts: the 1^st^ part denotes the individual code/GenBank Accession Number, the 2^nd^ part denotes the country of origin, and the 3^rd^ part denotes the date of sample collection (as in Figure 2). For Mexico samples, all of which were generated in this study, each location code comprises a unique two-digit code followed by an underscore and then the location in the Yucatan Peninsula, as given in Table S1. Sequences (dots) are also coloured based on location: red for Yucatan, Mexico (this study), green for elsewhere in N and S America, purple for Europe and yellow for East Asia. Black dots represent putative, unsampled haplotypes whilst a bar represents 1 base difference. GenBank Accession codes for the 11 Mexican sequences generated in this study are given in Table S5). Haplotype network constructed and visualized using PopART (https://popart.maths.otago.ac.nz/) (Leigh & Bryant, 2015).
